# Editorial: The Mononuclear Phagocyte System in Infectious Disease

**DOI:** 10.3389/fimmu.2019.01443

**Published:** 2019-06-26

**Authors:** Geanncarlo Lugo-Villarino, Céline Cougoule, Etienne Meunier, Yoann Rombouts, Christel Vérollet, Luciana Balboa

**Affiliations:** ^1^CNRS, UPS, Institut de Pharmacologie et de Biologie Structurale, IPBS, Université de Toulouse, Toulouse, France; ^2^International Associated Laboratory, CNRS “IM-TB/HIV” (1167), Toulouse, France; ^3^IMEX-CONICET, Academia Nacional de Medicina, Buenos Aires, Argentina

**Keywords:** monocytes, macrophages, dendritic cells, infection, pathogen, microbe, inflammation

The term “Mononuclear Phagocyte System” (MPS) was introduced by van Furth and Cohn in 1968 to describe a group of leukocytes that shared phenotypic features (e.g., a single nucleus) and biological functions (e.g., phagocytosis) (1). This term served originally to characterize bone marrow progenitors, blood monocytes, and tissue macrophages, under the assumption that it was a linear progression from progenitor to monocyte, and from monocyte to macrophage. Upon the discovery of dendritic cells (DC) in 1973 by the late Nobel Laureate, Ralph Steinman, and subsequent inclusion of this cell type as part of MPS in the late 1970s, the term “MPS” undertook a specialized function for processing and presenting antigen to activate lymphocytes (2). Monocytes, DCs, and macrophages became referred to as antigen-presenting cells (APC). Today, beyond serving as primordial APCs, these cells are also known to play roles in thermogenesis, tissue development, and organ function, maintenance of homeostasis, microbiota interactions, innate immunity against pathogens, inflammation and its resolution, and wound healing and tissue repair, among others (3). Also, it is now clear that monocytes, DCs and macrophages, are not homogenous populations (4). Recent conceptual advances concerning the MPS ontogeny and development have shattered the traditional view of DCs and macrophages as linear derivates and functional variations of monocytes (5). It is predicted that the incorporation of new technologies (e.g., mass cytometry, single-cell RNA sequencing) along with the progress in imaging capacities, will continue to unveil cellular heterogeneity and behavior in different tissues, differentiation trajectories, and the identification of novel immune functions, within the mouse and human MPS (5). Therefore, as the MPS field continues its unrelenting progress, it is important to regularly revisit the MPS conceptual framework in health and disease.

This is precisely what we accomplished through the collection of articles in this research topic entitled “The Mononuclear Phagocyte System in Infectious Disease”. As its title indicates, there are a total of 60 articles (26 original research and 34 literature reviews) written by 379 authors and published within this eBook dealing with multiple aspects of host-pathogen interaction. The tenet of this article collection is that a better understanding of MPS in infection will yield insights for development of therapeutics to enhance antimicrobial processes or dampen detrimental inflammation, for the benefit of the host. We believe that the contributions made to this topic will serve as a platform for discussion and debate about relevant issues and themes in this field.

## Introduction to MPS Biology and Function

For didactic purposes, we begin this collection with a series of “introductory” articles where readers can find general informations about MPS biology and functions in the context of infectious and inflammatory diseases.

Addressing cell-intrinsic functions, Uribe-Querol and Rosales discuss the influence on phagocytosis by microbial pathogens. Following an overview of phagocytosis and the antimicrobial factors employed by MPS, they summarize the microbial strategies used to inhibit phagocytosis at the level of ingestion, phagosome formation, and maturation, including microbial escape from these vesicles. Münz takes on autophagy in the context of viral infection. He describes recent evidence for how autophagy proteins regulate endocytosis and exocytosis in MPS cell activation, viral replication, and antigen presentation. Reciprocally, Bah and Vergne focus on autophagy in the context of bacterial infections. They discuss multiple functions of autophagy proteins in conferring cell defense against bacteria, and discuss how some pathogenic bacteria manipulate autophagy. Together, these authors argue that a better understanding of autophagy proteins might yield new therapeutic approaches against pathogenic infections. In terms of factors involved in the inflammatory response against pathogens, Weber et al. cast light on Bruton's tyrosine kinase (BTK). Beyond its central role as a mediator of B cell receptor signaling, BTK is crucial in the recognition of infectious agents, cellular maturation, and recruitment, and inflammasome regulation. Thus, it will be beneficial to improve our understanding of BTK biology not only as a kinase, but also as a scaffold protein, within the MPS. As a final point in cell-intrinsic properties, Karaji and Sattentau take on the efferocytosis of pathogen-infected cells, which is the process of clearing of unwanted cells by MPS. After a description of pathogen-triggered cell death and the signals inducing efferocytosis, they discuss how it controls different pathogenic infections and generates immune responses. Conversely, pathogens have evolved ingenious strategies to subvert efferocytosis, thus opening avenues to block immune evasion and pathogen persistence. The importance of efferocytosis in the resolution of inflammation is highlighted in the study by Lutati et al., who identified a neutrophil-derived glycoprotein lactoferrin (Lf) that triggers resolution-phase macrophages. Indeed, they demonstrate that Lf promotes the conversion of pro-inflammatory into pro-resolution macrophages that have enhanced efferocytotic capacity to clear apoptotic neutrophils.

We continue our introductory articles to address pathophysiological aspects of the MPS. Dorhoi and Du Plessis take on the monocytic-like myeloid-derived suppressor cells (M-MDSC), which inhibit innate and adaptive immune cell activation, proliferation, viability, trafficking, and cytokine production, to promote peripheral tolerance against unwanted inflammation during pathogenic infection. However, M-MDSC also serve as reservoirs for pathogens favoring their survival and limiting optimal host responses, highlighting the need to better understand their genesis and biological functions in chronic infections. Silva et al. focus on the role of E-Selectin ligands to home blood-borne MPS cells into infection sites. Insights to the structural and functional properties of E-Selectin ligands in MPS cells are likely to improve the efficiency of immune responses and ameliorate immunopathology derived from chronic inflammation. Within the context of cell migration, Cougoule et al. reveal the importance of podosomes (actin-rich structures found specifically on the basal membrane of MPS cells) for the capacity of human DCs to penetrate dense microenvironments. This study describes the influence of pathogen-derived signals on podosome formation and dynamics, thus dictating the mode of migration of DCs toward infectious sites. Da Silva et al. provide an overview on Peyer's patch MPS in health and disease. This includes MPS cell diversity and specificity, anatomical localization, functions, interaction with microbiota and generation of humoral immunity, and their role in multiple infectious contexts.

Recent advances in MPS biology in the cardiovascular system is the subject for the review by Sanmarco et al. MPS cells play roles in cardiac inflammation and infection, such as in Chagas cardiomyopathy, viral myocarditis, bacterial infections, sterile inflammation, among other disorders. The authors highlight the importance of dissecting the signaling pathways and molecules modulating MPS functions during infection. Lastly, Poulin et al. tackle the myeloid postulate of immune paralysis in sepsis by focusing on the well-known disappearance of circulating DCs. Beyond the description of the DC ontogeny and functions, they discuss about novel myelopoiesis-based therapeutic targeting MPS cells in sepsis. As described above, sometimes infection may lead to uncontrolled inflammation causing immunopathology. In their study, Peralta-Ramos et al. address the role of type-I interferons (IFN-I) in establishing and developing neuroinflammatory diseases. Upon systemic inflammatory challenge, they show that IFN-I promotes the recruitment of inflammatory monocytes into the brain, which subsequently induces local T cell activation leading to immunopathology.

## MPS Biology in Different Infectious Diseases

This section addresses the interaction of human and mouse MPS with different types of pathogens: bacterial, viral, fungal, and parasitic.

### Tuberculosis Disease

Reflecting the expertise contained within our editorial group, we provide a robust collection of articles that address MPS cell interaction with *Mycobacterium tuberculosis* (Mtb), the etiological agent responsible for tuberculosis. Within the context of cell heterogeneity, Sampath et al. discuss how monocyte subsets participate as effector and cell targets contributing to the control and pathogenesis of Mtb, respectively. Likewise, Marakalala et al. review macrophage heterogeneity in Mtb tropism, granuloma formation and maintenance, activation and product secretion during infection, foam cell formation, and caseation, among other aspects. Contrary to classical activation of pro-inflammatory macrophages with IFNγ, there are other immune signals that activate macrophages toward alternative phenotypes, such as interleukin (IL)-4, IL-10, and IL-6. In their study, Lugo-Villarino et al. address the role of the C-type lectin receptor (CLR) DC-SIGN, a marker for IL-4-activated macrophages, in the response against Mtb. While this CLR helps Mtb to parasitize macrophages, they describe a parallel role as a molecular switch to turn off the pro-inflammatory response to prevent unwanted immunopathology associated to tuberculosis. Using an acellular fraction of tuberculous pleural effusions as a physiological source of local factors released during Mtb infection, Genoula et al. characterize how the IL-10/STAT3 axis induces foam cell differentiation by activating the enzyme acyl CoA:cholesterol acyl transferase, contributing to our understanding of how alterations of the host metabolic pathways may favor pathogen persistence. Excessive activation toward a macrophage subset may often lead to susceptibility to pathogen invasion or immunopathological disorders. Employing a mouse model targeting the deletion of the suppressor of cytokine signaling 3 (SOCS3) specifically in macrophages, Schmok et al. demonstrate how SOCS3 prevents macrophage activation driven by IL-6, increasing susceptibility to Mtb replication in lung macrophage.

Continuing with the interaction between macrophages and Mtb, Queval et al. review the molecular events governing the bacilli survival, and how it is able to transform macrophages into survival and dissemination vessels. One of these aspects is the regulation of metabolism that is critical in shaping macrophage activation and immune functions. Teng et al. write about mycobacterial lipid-based tactics to manipulate host metabolism. They address the emerging roles of lipids in the complex host–pathogen relationship, and discuss recent methodologies used to assess lipid dynamics during infection (Teng et al.). Within this context, Oldenburg et al. report how mycobacterial phenolic glycolipids (PGL) decrease the TLR-4-dependent signaling cascade, resulting in the low production of pro-inflammatory signals in macrophages. The authors show that PGL-1 targets the posttranscriptional decrease of TIR-domain-containing adapter-inducing interferon-β (TRIF) protein level, an important component of the TLR-4 signaling pathway. Likewise, Chavez-Galán et al. describe how Lipoarabinomannan affects the T-cell immunoglobulin and mucin domain 3 (TIM3)/galectin (GAL)9 pathway by targeting GAL9 expression. This is important because the interaction between TIM3 and GAL9 was shown to promote microbicidal activity against Mtb. Another strategy used by Mtb is to control the activity of host lysosomal cathepsins in macrophages. Pires et al. demonstrate that Mtb upregulates miRNAs such as miR-106b-5p, which binds to the 3′-untranslated region of the mRNA for cathepsin S, thus preventing its protein expression and consequently increasing Mtb survival.

Beyond tuberculosis, we include a study on “nutritional immunity” in the context of *Salmonella enterica* serovar Typhimurium infection. This is a host defense strategy against infection that relies on the sequestration of essential molecules, such as iron, to prevent pathogen growth. However, pathogens evolved counter strategies to deal with nutritional immunity. In their study, Willemetz et al. demonstrate that *Salmonella* modulates macrophage iron homeostasis to favor its access to high intracellular content of iron by downregulating the iron exporter ferroportin (FPN). Intriguingly, FPN is repressed through an iron and hepcidin-independent mechanism, suggesting the cellular iron is indispensable for the growth of *Salmonella* inside the macrophages (Willemetz et al.). It will be interesting to find out whether this is a general host response observed with other intracellular pathogens infection, such as Mtb.

As we move forward in understanding the host and Mtb interactions, there are various aspects that require further refinement. For example, Donovan et al. address the role of IFN-I in tuberculosis. In their review, the authors argue the need to identify the myeloid source for IFN-I production during Mtb infection *in vivo*; characterize the roles for individual members of the IFN-I family; and define whether blood gene expression signatures in active tuberculosis are a natural consequence of Mtb infection in humans or an indication of disease susceptibility. Another field requiring further elucidation is MPS immunometabolism. Using novel genetic mouse models to delete acetyl-CoA carboxylase (ACC) 1 and 2 specifically in DCs, macrophage or T lymphocytes, Stuve et al. clearly demonstrate that MPS cells do not require these enzymes for the control of mycobacteria infection. By contrast, targeted deletion in T cells results in susceptibility to Mtb. One novel approach to more deeply investigate how Mtb modulates the host response is to perform mass spectrometry (MS)-based proteomics on infected cells. Specifically, Hoffmann et al. summarize the progress made in determining the protein composition of mycobacteria-containing vacuoles, which has already shown, for example, how Mtb prevents the fusion of late endosomes and lysosomal compartments. The authors argue this approach will promote the development of novel host-directed therapies, particularly against the emergent drug-resistant Mtb strains. Likewise, Pahari et al. propose different strategies in their review to reinforce the functional aspects of the MPS to achieve better control of Mtb. They discuss multiple molecules to boost the innate immune system to enhance the emergent field of immunotherapeutics. For instance, Zhang et al. show in their study that the herbal medicine, baicalin, induces autophagy activation in Mtb-infected macrophages leading to a better control of intracellular Mtb. At the same time, baicalin also regulates the inflammasome-dependent activation of IL-1β, suggesting this herbal medicine also controls unnecessary inflammation derived from infection (Zhang et al.). An area that requires improvement is the assessment of biomarkers according to the different states of disease progression and severity. In their study, Ranaivomanana et al. provide a biomarker signature that is different between patients with pulmonary and extrapulmonary tuberculosis, and they correlate it in human macrophages infected *in vitro* with clinical strains representative for each tuberculosis disease type. Finally, inter-species comparisons are helpful not only to advance our understanding of the MPS, but also necessary to deal with infectious diseases afflicting in domestic animals. In bovines, the *Mycobacterium avium* subspecies *paratuberculosis* (*Map*) causes a chronic intestinal infection known as paratuberculosis, which leads to significant economic loss to livestock industries. One of the problems is that early host-pathogen interactions in bovines remain poorly understood. In their study, Baquero and Plattner report that γδ T cells influence differentiation, maturation, and functional aspects of bovine monocytes during *Map* infection. The authors argue that modulation of the γδ T cells/MPS axis represents a viable vaccination/therapeutic strategy to generate a strong adaptive immunity to counteract paratuberculosis in bovines (Baquero and Plattner).

Collectively, this series of articles promote the importance of MPS in the tuberculosis context, and it highlights its therapeutic potential yet to be exploited to control this disease.

### HIV and Other Viral Diseases

Reflecting another area of expertise within our editorial group, the following article collection addresses MPS cell interaction with the human immunodeficiency virus (HIV). We start with a review article by Rodrigues et al., who provide an in-depth description of HIV-macrophage interaction during the various stages of the virus life cycle. This is complemented by another review article from Merino et al. discussing the contribution of monocytes and macrophages to the infection and progression of HIV and SIV, a closely related HIV-like virus that causes disease similar to AIDS in monkeys and apes. The macrophage interaction with HIV is important because it influences the adaptive immune response. Trifone et al. demonstrate the role of macrophage migration inhibitory factor (MIF)/CD74 axis in HIV pathogenesis. They show that HIV-1 infection of CD74^+^ macrophages results in a pro-inflammatory environment in the presence of MIF, which predisposes unactivated CD4^+^ T cells to HIV-1 infection (Trifone et al.).

Another way the MPS participates in HIV pathogenesis is through cell-to-cell transmission of the virus, including through interactions with lymphocytes. In their review, Bracq et al. provide a description of the different mechanisms that occur during cell-to-cell transmission of HIV, such as intercellular structures and membrane protrusions, immunological synapses, efferocytosis of dying cells, and cell-to-cell fusion. Concerning intercellular structures and membrane protrusions, Dupont et al. focus their review on tunneling nanotubes (TNT), which are long membranous dynamic structures and a novel route for cell-to-cell communication. They argue TNT serve as major “corridors” for viral to spread efficiently to bystander cells and remain undetected by the immune system (Dupont et al.). As a strategy to hijack cell-to-cell communication between MPS cells, Martinez-Picado et al. write about the lectin receptor Siglec-1/CD169 that facilitates the binding of HIV-1 without internalization and, consequently, the infection of bystander cells. In their review, they discuss how working with human cohorts lacking Siglec-1 provides a unique understanding of the role this receptor plays in viral pathogenesis *in vivo*.

An important issue of HIV-positive patients is their susceptibility of co-infection with other pathogens. For example, patients living with HIV are more likely to die from tuberculosis than HIV-negative people. Addressing this issue, Gupta-Wright et al. developed a flow cytometry-based fluorescent reporter of phagosomal oxidase activity to demonstrate impaired superoxide burst activity in the phagocytes of hospitalized HIV-positive patients with tuberculosis. The authors promote the use of this assay to assess the immune competence of co-infected patients in clinical settings, including the activation state of MPS cells. Another complication in HIV-positive patients is susceptibility to opportunistic pathogens such as non-typhoidal *Salmonella enterica* serovar Typhimurium, which is the most common cause of bacterial bloodstream infections in these patients. In their review, Lê-Bury and Niedergang describe how macrophage function is impaired upon HIV infection, discuss factors that make invasive Salmonella Typhimurium specific for HIV pathogenesis, and provide reasons for why these bacteria are well-suited to invade the HIV-infected host.

This section closes with an overall look at the MPS in the context of other viral infections. Pombo and Sanyal review different mechanisms of altering lipid metabolic pathways during infection by flaviviruses, and they focus on cholesterol and fatty acid biosynthesis. Intriguingly, these events can promote an advantage for invading viruses to support replication, and thus they can be modulated for the benefit of the host as a mean to fight infection. Since the Human Respiratory Syncytial Virus (hRSV) is responsible for bronchiolitis, pneumonia, recurrent wheezing and asthma, Bohmwald et al. provide a review on the role of the lung MPS during hRSV infection and their involvement in its pathogenesis. This is critical given that hRSV causes functional impairment of the MPS. Next, Mulder et al. use the Lymphocytic Choriomeningitis Virus (LCMV), of the family Arenaviridae, an etiological agent for human acute aseptic meningitis and grippe-like infections, to understand how different macrophage subsets are able to control viral infection and activate the adaptive immune response in the mouse model. In their study, they show that IL-4-activated macrophages do not support CD8^+^ T-cell proliferation and effector functions during virus infection, as opposed to other macrophages activated by stimuli such as IFN-γ, for example. As part of the inter-species comparisons included in this eBook, Zhang et al. assess the impact of virulent Newcastle Disease Virus (NDV) infection in the macrophage compartment in chickens. Indeed, NDV causes Newcastle disease that is highly contagious and fatal in birds that dramatically affects the global domestic poultry production. The authors describe that the macrophage compartment becomes susceptible to viral replication due to inhibition of TLR-7-dependent signaling, which is avoided by pre-treatment with TLR-7 ligand (Zhang et al.).

Together, this series of articles promote the importance of MPS in viral infection, highlighting the need to better understand the interaction of MPS cells with viruses and the mechanisms involved in viral subversion and evasion strategies.

### Fungal and Parasitic Diseases

This eBook continues with an overall perspective of the MPS in the context of parasite and fungal infection. Human parasites are divided into endoparasites (cause infection inside the body) and ectoparasites (cause infection superficially within the skin). Parasitic worms (helminths) are endoparasites that infect ~24% of the entire human population. For this reason, Motran et al. provide a discussion of the recognition and modulation of the MPS during helminth infections. In particular, they address excretory-secretory products resulting from helminth recognition by DCs and macrophages. With regards to recognition of helminths, van Die and Cummings shed light on the macrophage Mannose Receptor (MR) in the MPS to that shapes the immune responses to these parasites, and they deliver an overview of the structural aspects of this receptor along with important functional implications. Another endoparasite that afflicts the human population and affects the MPS is *African trypanosomosis*, which causes a debilitating disease known as “sleeping sickness.” In their review, Stijlemans et al. deliver a discussion of how factors derived from this parasite and the MPS contribute to trypanosomosis-associated anemia development, along with intervention strategies partially targeting the MPS to alleviate this disease. This is complemented by another review by Holzmuller et al., who focus on how trypanosomatid excreted-secreted or host molecules affect the metabolic balance between nitric oxide synthase/arginase in the activation of macrophages, and their overall impact on the pathogenesis of this disease. Within the metabolic context, Rojas-Márquez et al. report that *Trypanosoma cruzi*, the causative agent for Chagas' disease, is controlled inside macrophages by the activation of the NLRP3 inflammasome and reactive oxygen species (ROS) production. Interestingly, they show that the metabolic checkpoint kinase mammalian target of rapamycin (mTOR) is activated by *T. cruzi* infection and it negatively regulates the NLRP3 activation, and its pharmacological inhibition restores the microbicidal properties of macrophages against this parasite (Rojas-Márquez et al.).

Leishmaniasis is a disease caused by protozoa endoparasites of the *Leishmania* order, affecting 4–12 million people in the world. It is manifested at the cutaneous, mucocutaneous or visceral level. In the context of cutaneous Leishmaniasis caused by *L. major*, Vellozo et al. show that, while this parasite promotes the pro-inflammatory activation of anti-parasitic macrophages, the all-trans retinoic acid (ATRA) therapy prevents this differentiation rendering susceptibility in the host. This is important because the ATRA treatment has been proposed as a therapy to differentiate immature myeloid cells into macrophages to boost immunity against tumors, suggesting an increased risk for endoparasite infections in such patients. In the context of visceral Leishmaniasis caused by *L. infantum*, Peres et al. address the role of Ecto-nucleotidases in the infection of human macrophages. They demonstrate ecto-nucleotidase activity of *L. infantum* is directly associated with infectivity of macrophages, which is blocked using antibodies targeting this enzyme, opening new therapeutic avenues against Leishmaniasis and other trypanosomitides infections (Peres et al.). Other forms of endoparasite are parasitic flukes, such as liver fluke disease that is caused by *Fasciola hepatica*. In their study, Carasi et al. investigate the role of heme-oxygenase-1 (HO-1) in the immunoregulation of DCs by *F. hepatica*. They show that the HO-1 expression favors this parasite infection, resulting in increased clinical signs and liver damage. This is associated with the upregulation of IL-10 that down-modulates the activation of DCs and macrophages. Indeed, the authors demonstrate that enzymatic inhibition of HO-1 leads to a decrease of IL-10 and an increase in the resistance of mice against infection, indicating that *F. hepatica* employs HO-1 to subvert the MPS (Carasi et al.).

Finally, ectoparasites can also be a burden to the human population even in developed countries. For example, tick-transmitted *Anaplasma phagocytophilum* is responsible for human granulocytic anaplasmosis, which is the third most common human vector-borne infection in USA causing lethality in 1% of patients. Here, Scorpio et al. report that impaired cytotoxicity against *A. phagocytophilum* is associated with a defect in APCs that express MHC class I and interact with innate and adaptive immune cells during infection, inferring that parasite-derived products may alter the MPS functions to circumvent CD8^+^ T cell cytotoxicity.

This section concludes with a brief overview of the role of MPS in mycosis, which are a public health problem afflicting humans; there is an estimated 1.6 million people who die each year of fungal infections. To understand the host-fungi interactions, fundamental research has turned partially toward the use of fungal cell wall polysaccharides due to their immunogenicity properties and potential to be therapeutic compounds in infectious disease. In their review, Camili et al. write about the complexity of MPS interaction with β-glucan, the most abundant fungal cell wall polysaccharide. It is argued that a better understanding of the biochemical and immunogenicity properties of the different fungal β-glucans (isolated from different sources) will be necessary to uncover novel cellular and molecular mechanisms of action, and to improve a rational use in the future as an adjuvant or therapeutic agent. One of the problems with blood stream fungal infection is the lack of an appropriate and rapid treatment. This is the main issue addressed by Leonor Fernades-Saraiva et al., who developed a combined classifier approach with distinct differential gene expression profiles across multiple studies to generate an exclusive lysosome-related gene expression as a blood monocyte-specific footprint for fungal infections. This is crucial because it is specific for fungal (as opposed to bacterial) infection and it may allow its rapid identification in the patient's blood. Within the context of therapeutic perspectives, Benmoussa et al. identify a novel compound P17, derived from ant venom, with the capacity to modulate macrophage differentiation toward an anti-fungal phenotype characterized by a signaling pathway dependent on the peroxisome proliferator-activated receptor gamma (PPARγ), Dectin-1 and MR receptors.

Taken in concert, while the MPS plays a key role in controlling and generating immunity against parasitic and fungal infections, these pathogens have also evolved strategies to circumvent and subvert the MPS. Understanding this complexity represents an opportunity for diagnostic, prevention, and therapeutic advances.

## Alternative and Novel Models

In this section, we offer a wider perspective of the MPS with a series of articles describing alternative animal and cellular models to highlight different approaches, limitations, and advantages to better define MPS functional properties that can be exploited for therapeutic and vaccination purposes.

With regard to animal models, the zebrafish (*Danio rerio*) model symbolizes complementarity with the human and mouse models. This is a unique platform to study the MPS interaction with pathogens *in vivo*, ranging from the single cell level to the whole organism. Advantages include: non-invasive real-time visualization of the MPS system, chemical and genetic tractability, and this model is a natural host of a great number of pathogens of bacteria, virus, parasite and fungal origin, among others. In their review, Yoshida et al. argue why zebrafish are a unique model to study macrophage-microbe interactions, focusing on recent developments in the context of bacterial and fungal infection. Furthermore, Boucontet et al. promote zebrafish as a model of superinfection with a virus (i.e., Sindbis virus) and a bacterium (i.e., Shigella). In their study, they uncover a virus-induced hyper-susceptibility to bacterial infection that is associated with defects in neutrophil functions. Thus, zebrafish offer an alternative model to better understand this phenomenon, and a platform for drug-testing and therapeutic-development to restore the host immunity (Boucontet et al.).

In terms of cellular models, use of the soil dwelling, social amoeba *Dictyostelium discoideum*, is already making important contributions to our understanding of cell-autonomous mechanisms in MPS cells. Dunn et al. provide a review of the advantages and relevance of *D. discoideum* to study the antimicrobial functions of phagocytosis and autophagy, and the microbial properties of the phagosome. Reciprocally, they also discuss microbial interference with these defenses, and address a variety of conserved microbial restriction factors in *D. discoideum* that are relevant to the mammalian MPS. Another cellular model likely to make an impact is that of embryonic derived, self-renewing human tissue-resident macrophages (Max Planck Institute, MPI cells), which mimic lung alveolar macrophages. In their study, Woo et al. perform characterization of the responses of MPI cells to Mtb infection, including recognition and ingestion of live Mtb, bacterial intracellular replication, induction of pro- and anti-inflammatory cytokines, microbicidal activity and elimination of bacteria, and accumulation of lipid droplets in the cytoplasm upon lipoprotein exposure. The authors conclude that MPI cells are a tractable cell model for tuberculosis research that will enhance our understanding of human alveolar macrophages (Woo et al.). Finally, Iakobachvili and Peters provide an outlook on the “Organoid Revolution” that is currently taking place in all fields of biology. They explain organoid technology, describe its current impact in infectious diseases, and discuss how it may be used to study MPS biology in infection (Iakobachvili and Peters). Altogether, these cellular models are compliant with the 3Rs (Replace, Reduce, Refine) initiative to reduce the use of animals to study the MPS, and provide a context to study cell-autonomous defenses independent of other immune responses.

## Summary

We compiled expertise of scientists of all career stages and from 23 countries and five continents to address recent progress, highlight critical knowledge gaps, foment scientific exchange, and establish conceptual frameworks for future MPS investigation in the context of infectious disease. We hope that the present MPS conceptual framework will enhance the reader's knowledge within the infectious disease context, and inspire new lines of investigation to further fuel the progress in this field.

## Author Contributions

GL-V wrote manuscript. CC, EM, YR, CV, and LB edited and contributed to the organization of the editorial article.

### Conflict of Interest Statement

The authors declare that the research was conducted in the absence of any commercial or financial relationships that could be construed as a potential conflict of interest.
